# Intergroup Relations During the Refugee Crisis: Individual and Cultural Stereotypes and Prejudices and Their Relationship With Behavior Toward Asylum Seekers

**DOI:** 10.3389/fpsyg.2020.612267

**Published:** 2020-12-18

**Authors:** Hege H. Bye

**Affiliations:** Department of Psychosocial Science, University of Bergen, Bergen, Norway

**Keywords:** stereotypes (social psychology), prejudice/stereotyping, intergroup behavior, refugee crisis, asylum seeker, stereotype content model

## Abstract

In this paper, I investigate intergroup relations between natives and asylum seekers during the European refugee crisis, and contribute to the reemerging methodological debate on the measurement of stereotypes and prejudices as individual and collective constructs. Drawing on data from the Norwegian Citizen Panel (NCP; *N* = 1,062), I examined how Norwegians stereotyped asylum seekers at the height of the refugee crisis and the emotional prejudices asylum seekers as a group elicited. By experimentally manipulating the survey question format, I examined whether and how stereotypes and emotional prejudices toward asylum-seekers differed depending on their measurement as individual or collective constructs. A subset of respondents (*n* = 228) had reception centers for asylum-seekers established in their local community during the crisis. These participants reported their behaviors toward the asylum seekers in their neighborhood. In this subsample, I investigated how individual facilitating and harming intergroup behavior was related to individual and collective conceptualizations of stereotypes and prejudices. The results showed that both low warmth and low competence stereotypes, as well as negative emotions toward asylum seekers, were rated as stronger when measured as collective as compared to individual-level constructs. In the individual condition, respondents reported feeling more admiration and sympathy than respondents in the collective condition attributed to others. Individual stereotypes and prejudices correlated systematically with individual facilitating and harming intergroup behaviors. The perception that others hold more negative stereotypes of asylum seekers, and the perceived anger and fear of others, did correlated with individual harming behaviors. Perceptions of others’ anxiety correlated negatively with facilitating behaviors. Implications and future directions for the conceptualization and measurement of stereotypes and emotional prejudices are discussed.

## Introduction

During the 2015/2016 refugee crisis, more than 1 million people sought refuge in Europe, the majority fleeing from the war in Syria (UNHCR, 2016). The way receiving populations respond to an influx of refugees may have far-reaching consequences, for example, by affecting the health and well-being of the incoming refugees and by impacting voting in local and national elections. Researchers across the social sciences have therefore strived to document and explain responses among receiving populations during and in the aftermath of the 2015/2016 crisis (Esses et al., 2017; Kotzur et al., 2017, 2019a; Bruneau et al., 2018; Dinas et al., 2019; Hangartner et al., 2019; Steinmayr, 2020). A range of factors – from the number of asylum seekers entering the country to political discourse and government policies – varied across receiving nations. In this paper, I contribute insights from Norway, one of the major receiving countries in the European Economic Area (EEA) relative to population size (Eurostat, 2016).

During the course of 1 year, Norwegian authorities responded to the refugee influx by establishing 259 reception centers for asylum seekers (asylum seeker centers; ASCs) in local communities across the country (Bygnes, 2020). For the asylum seekers, many of whom were later granted refugee status, being lodged in an ASC provided the first local context for cultural contact and acculturation. Local and national environments may be adaptive or restrictive for asylum seekers’ acculturation processes (Donà and Young, 2016; Bruneau et al., 2018), depending on factors such as settlement policies, economic support, and access to healthcare. From a social psychological perspective, central features of reception contexts are the stereotypes, prejudices and intergroup behaviors of members of the receiving communities (Esses et al., 2017).

The purpose of this paper is two-fold. With the Stereotype Content Model (SCM) and the Behavior from Intergroup Affect and Stereotypes (BIAS) Map (Fiske et al., 2002; Cuddy et al., 2007) as a theoretical framework, I address Norwegians’ stereotypes, prejudices and intergroup behaviors toward asylum seekers at a time (March 2016) when the population had witnessed an unprecedented number of asylum seeker arrivals, as well as the recent and rapid establishment of ASCs across the country. Thus, this paper contributes to the stream of research documenting receiving populations’ responses to the 2015/2016 refugee influx in Europe.

Second, there is a reemerging debate about how stereotypes and emotional prejudices should be conceptualized and measured (Stangor and Schaller, 1996; Findor et al., 2020; Kotzur et al., 2020). Should researchers focus on individuals’ personally held perceptions of social groups, and the individually experienced emotions toward them? Or, should we ask people about their perceptions of the broadly shared views of social groups within society and how “most people” feel toward other groups? By experimentally varying response instructions, I compare these two approaches to the measurement of stereotypes and prejudices, and contextualize my substantive findings on intergroup relations between natives and asylum seekers within this ongoing methodological debate.

### The SCM and the BIAS Map

This work starts from the SCM (Fiske et al., 2002) and its extension into the BIAS Map (Cuddy et al., 2007). A core tenet of the SCM is that the stereotype content associated with social groups can be organized along the dimensions of warmth and competence. Whereas perceived warmth is rooted in perceptions of others’ intent toward the self or the ingroup (i.e., friend or foe?), perceived competence is rooted in perceptions of others’ capabilities to act on their intentions. There are social structural relationships between groups underlying these perceptions; perceived status (e.g., power and economic resources) predicts perceived group competence, and perceived competition predicts (lower) group warmth (Fiske et al., 2002).

Another key aspect of the SCM is that perceptions of groups’ warmth and competence interact in eliciting specific emotional prejudices. Groups stereotyped as high in both warmth and competence elicit feelings of admiration and pride, whereas groups stereotyped as low in warmth and competence elicit disgust and contempt. Ambivalently stereotyped groups elicit envy and jealousy in the case of high competence and low warmth stereotypes, and pity and sympathy in the case of groups stereotyped as high in warmth and low in competence (Fiske et al., 2002; Cuddy et al., 2007).

Extending the SCM to include intergroup behaviors in the BIAS Map, Cuddy et al. (2007) argued that perceptions of warmth are associated with active behavioral tendencies of facilitation (high warmth) and harm (low warmth). Perceptions of competence are associated with passive behavioral tendencies (passive facilitation and passive harm). The active–passive dimension of facilitating and harming behaviors separates behaviors enacted with concentrated effort and intention (active), from those that require less effort and intention (passive). Finally, specific emotional prejudices are hypothesized to mediate the effect of stereotype content (warmth, competence) on intergroup behavior. Both Cuddy et al. (2007) and later studies have generally found support for the relationships between stereotype content, emotional prejudices and intergroup behaviors outlined in the BIAS Map, albeit with some variation in findings pertaining to the mediation hypotheses (see Bye and Herrebrøden, 2018, p. 1080–1082 for a review).

**Table 1:** Intergroup behaviors directed at asylum seekers and the ASC. Rotated principle components solution.

Item	Component loadings	
	Facilitation	Harm
Participated in voluntary work to help the asylum seekers	**0.90**	0.00
Initiated activities for the asylum seekers	**0.89**	0.09
Become friends with some of the asylum seekers	**0.84**	0.10
Said hello to some of the asylum seekers	**0.77**	−0.03
Given gifts to the asylum seekers’ center	**0.75**	−0.07
Written something positive about the asylum seekers’ center in the comments area or similar, without giving my name	**0.67**	0.44
Written something positive about the asylum seekers’ center using my full name in social media or similar	**0.63**	0.17
Participated in protests to stop the asylum seekers’ center being set up	0.12	**0.87**
Taken the initiative to protest to stop the asylum seekers’ center being set up	0.14	**0.84**
Written something negative about the asylum seekers’ center using my full name in social media or similar	0.32	**0.78**
Written something negative about the asylum seekers’ center in the comments area or similar, without giving my name	0.38	**0.77**
Avoided the center and the asylum seekers as far as possible	−0.21	**0.62**
Tolerated the establishment of the asylum seekers’ center	0.34	**−0.52**

Bold values indicate which component each item had the strongest loading on.

The focus of the BIAS Map is the uniquely human emotions of admiration, pity, envy, and contempt. However, Cuddy et al. (2007) also investigated the role of the primary emotions anger and fear in the stereotype – behavior relationship. They found that both anger and fear correlated negatively with warmth and positively with active harming behaviors. Anger also correlated negatively with active facilitation.

### Refugees and Asylum Seekers in the Stereotype Content Model

In a review of the extensive literature on public perceptions of refugees, Esses et al. (2017) highlighted several important findings regarding receiving populations’ reactions to asylum seekers and refugees. They describe prevalent negative attitudes and perceptions documented by public opinion polls across Europe and the United States in response to the refugee crisis – including associating refuges with terrorists, beliefs that refugee claimants are bogus, and concerns that refugees pose economic and cultural threats. They also describe the public discourse on refugees as increasingly dehumanizing (Esses et al., 2017). This is important because dehumanization has been linked with emotions of contempt and the absence of admiration (Esses et al., 2013), as well as support for anti-refugee policies and anti-refugee behaviors (Bruneau et al., 2018).

Connecting these findings to the SCM and the BIAS Map, the predictions that follow are that (a) asylum seekers as a group will be perceived as low in warmth because they are perceived to pose economic and cultural threats, (b) asylum seekers as a group will be perceived to be low in competence due to their limited power and resources in the country of reception, (c) asylum seekers as a group primarily elicit feelings of contempt, disgust, anger and fear, and (d) as a consequence face active and passive harming responses from native majority members.

There are still few studies of asylum seekers or refugees within the SCM. Notable exceptions are studies focusing on the German context in the wake of the refugee crisis (Kotzur et al., 2017, 2019a,b; Froehlich and Schulte, 2019; Wyszynski et al., 2020). These studies show diverging results. In line with the predictions outlined above, one set of studies showed that refugees as a generic category, as well as closely related groups (i.e., Syrian immigrants, Afghan immigrants, Syrian refugees, and Afghan refugees), were stereotyped as low to moderate in warmth and low in competence (Froehlich and Schulte, 2019; Kotzur et al., 2019a; Wyszynski et al., 2020).

Contrary to the predictions outlined above, the other set of studies showed that asylum seekers and refugees were stereotyped as moderate to high in warmth and moderate in competence (Kotzur et al., 2017, 2019b; Study 1a, 1b, and Study 2). Moreover, Kotzur et al. (2019b, Study 1b and Study 2) found that asylum seekers elicited little contempt, and moderate to high levels of admiration and pity, and that participants were generally willing to engage in solidarity based collective action on behalf of asylum-seekers, a form of active facilitation (Study 2). Similarly, Kotzur et al. (2017) found that Germans reported feeling very little contempt, anxiety, and anger toward refuges and asylum seekers, but moderate to high levels of pity and admiration. Their respondents also reported high to moderate levels of passive and active facilitation, and very low levels of harming behaviors.

The divergence in the substantive findings from these sets of studies is intriguing – especially because they are conducted in the same national context, within a time frame of just a few years, and with similar types of samples (mostly university students). The key difference between the two sets of studies is the instructions to the participants: in the first set of studies participants were asked to indicate how warm and competent the groups were as perceived by most people in society/Germany, and in the second set, they were asked to provide their personal views, emotions and behaviors.

### Stereotypes and Prejudices as Cultural and Individual-Level Constructs

In Fiske et al.’s (2002) formulation of the SCM, stereotypes were defined and measured as socially shared and consensual phenomena within a culture; participants were asked not to give their personal views, but to report the views of the American society. Similarly, the first operationalization of emotional prejudices in the SCM and BIAS Map focused on how, from the perspective of society, various social groups made the respondents’ group, or “people in America,” feel^1^ (Fiske et al., 2002; Cuddy et al., 2007). This focus on shared, group-level stereotypes and group-level emotional prejudice has its roots in a long tradition of conceptualizing stereotypes as collective phenomena (Katz and Braly, 1933; Blumer, 1958; Stangor and Schaller, 1996). Asking about what other people believe and feel about social groups is also argued to limit the degree of social desirability bias in people’s responses (Fiske et al., 2002; Cuddy et al., 2007). In contrast to the original formulation, and as described in the case of asylum seekers and refugees above, other researchers drawing on the SCM explicitly address stereotypes as personal beliefs (Kotzur et al., 2017, 2019b) and examine individual emotions associated with social groups (e.g., Becker and Asbrock, 2012; Kotzur et al., 2019b). This individual approach also has a long history in social psychology (Stangor and Schaller, 1996). Until recently (Findor et al., 2020; Kotzur et al., 2020) however, these two approaches to the measurement of the constructs in the SCM had not been systematically compared. As illustrated by the results pertaining to the social perception of asylum seekers/refugees detailed above, varying the instructions may produce results that have diverging substantive interpretations.

Kotzur et al. (2020) conducted three experimental studies to assess how varying response instructions impacted on the placement of social groups in the SCM space. They found that instructions to provide one’s personal view lead to more positive views on the deprecated dimension for ambivalently stereotyped groups (i.e., on the competence dimension for groups stereotyped as warm but incompetent, and on the warmth dimension for groups stereotyped as competent but cold) as compared to the instructions to take society’s perspective. For groups stereotyped as low on both warmth and competence in the collective condition, personal views were more positive on both the warmth and the competence dimensions. These findings are important because they begin to answer the question of how response instructions impact on the placement of social groups in the warmth × competence space. However, Kotzur et al. (2020) did not test the impact of varying response instructions on the emotional prejudices reported toward groups, and which implications differences in response instructions may have with respect to the prediction of intergroup behaviors.

Findor et al. (2020) investigated the effect of response instructions on the full BIAS map model. Their target groups were two ethnic minority groups in Slovakia – Roma and Hungarians. I focus here on the Roma, the stigmatized minority of the two groups. In line with Kotzur et al. (2020), Findor et al. (2020) found that stereotype ratings of the Roma were more positive on both the warmth and competence dimensions in the individual condition. Moreover, they found that the reported levels of contempt and envy toward the Roma were higher, and admiration and pity lower, in the collective condition as compared to the individual condition. In the collective condition, the Roma were perceived to elicit substantially more active and passive harm, than in the individual condition. These results illustrate that response instructions may impact not only stereotype ratings, but also ratings of emotional prejudice and intergroup behaviors. What remains an open question, however, is how perceptions of shared, collective stereotypes and prejudices relate to individual intergroup behaviors.

According to Stangor and Schaller (1996, p. 5).

…the pivotal point of distinction between individual and collective approaches [to stereotypes] lies in the assumed importance of shared social beliefs, above and beyond the importance of individual beliefs, as determinants of social behavior. This distinction is particularly important for a complete understanding of stereotypes and stereotyping.

In the development of the BIAS Map, the measurement of intergroup behaviors was aligned to the measurement of stereotypes and emotional prejudice as shared, collective phenomena. Respondents were asked to indicate how people in America generally behave toward various social groups (Cuddy et al., 2007). Cuddy et al. (2007, p. 644) argued, consistent with their collective approach, that “even when individuals personally reject stereotypes that are prevalent in their cultures, they know and often cannot help but be affected by them. (…) exposure to (even without endorsement of) cultural stereotypes considerably affect reactions to outgroups.” However, they also recognize that “societal prejudices do not always equal personal prejudices. We do not yet know how the perspective of the perceiver will affect the BIAS map’s relationships at the personal level, a central question for future research” (p. 644). This question may be approached in different ways, one of them is to compare the relationships that stereotypes and emotional prejudices measured as individual and collective constructs exhibit with individuals’ own intergroup behaviors.

### The Present Study

In the present study, I build on and extend recent research on intergroup relations between host populations and asylum seekers/refugees within the SCM framework (Kotzur et al., 2017, 2019a,b; Froehlich and Schulte, 2019; Wyszynski et al., 2020) and the work of Kotzur et al. (2020) and Findor et al. (2020) on the impact of response instructions. Specifically, I compare the stereotype content and emotional prejudices associated with asylum seekers as a group under instructions to indicate either one’s personal opinion or to take the perspective of others in society.

With respect to stereotype ratings, previous research on the perceived warmth and competence of refugees and asylum seekers (Kotzur et al., 2017, 2019a,b; Froehlich and Schulte, 2019; Wyszynski et al., 2020) and the results of Kotzur et al. (2020) and Findor et al. (2020) suggest that asylum seekers will be rated as low in both warmth and competence in the collective condition, and specifically that they will be perceived as comparatively warmer and more competent in the individual condition.

Hypothesis 1. Asylum seekers are perceived as higher in warmth and competence when stereotypes are conceptualized as individual beliefs compared to perceived collective beliefs.

A core assumption in the SCM and the BIAS Map is the systematic relationship between stereotype content and emotional prejudice (Fiske et al., 2002; Cuddy et al., 2007). It follows from this principle that if the stereotype content associated with asylum seekers differs meaningfully across response instructions (e.g., from low/moderate warmth and low competence in the collective condition to high warmth and moderate competence in the individual condition, as would be expected based the research discussed above), the emotional prejudices elicited in the two conditions will differ. Specifically, I expect that:

Hypothesis 2. Asylum seekers as a group will elicit more contempt, disgust, anger and fear in the collective than the individual emotions condition.
Hypothesis 3. Asylum seekers as a group will elicit more pity, sympathy, admiration, and pride in the individual compared to the collective emotions condition.

With respect to intergroup behaviors directed toward asylum seekers, reactions in Norway to the refugee influx in 2015 included both facilitating (e.g., donating money and volunteering to help the asylum seekers) and harming behaviors (e.g., protesting the establishment of ASCs and in one extreme case setting fire to a planned ASC facility; Bygnes, 2020). Whereas some behaviors, such as protesting political decisions on social media or donating money, may be enacted irrespective of the presence of asylum seekers in the local environment, other behaviors (e.g., greeting asylum seekers or avoiding them) are primarily relevant for people who live in communities that host asylum seekers. In this paper, I focus on the intergroup behaviors of a subset of respondents who had an ASC established close to where they lived. In this group, I compare the extent to which the two different conceptualizations of stereotypes and emotional prejudices are related to respondents’ facilitating and harming behaviors toward asylum seekers in their local communities.

## Materials and Methods

### Design

I conducted a survey experiment employing a between–groups design. Participants were randomly asked to indicate their personal views and emotions (individual condition, *n* = 525), or the views and emotions of people in Norway in general (collective condition; *n* = 537) on asylum seekers as a group. The experiment was embedded in a larger survey module including questions about the establishment of reception facilities for asylum seekers in the participants’ neighborhood, and how respondents had behaved toward asylum seekers hosted in ASCs established in their local community in response to the crisis.

### Participants and Procedure

The experiment was embedded in the Norwegian Citizen Panel (NCP; Ivarsflaten et al., 2019). This is an online panel, where a random sample of the Norwegian population answers questions on a range of issues (e.g., climate change, politics, and immigration) two to three times a year. The sample is drawn from the Norwegian Population Registry, and all inhabitants above the age of 18 have an equal probability of being invited to the panel. Due to different rates of participation, there are some groups (e.g., young men with low levels of education) that are underrepresented (or overrepresented) in the sample compared to the population. For details on representativeness, please see the NCP methodology Report for wave 6 (Skjervheim and Høgestøl, 2016).

In wave 6 of the NCP (fielded March 1–19, 2016), 1,256 respondents were randomly assigned to be in the subpanel which contained the experiment. Due to the focus on asylum seekers as a target group, individuals were excluded if they indicated being citizens of another country, had an immigrant background, or declined to answer the citizenship/immigration questions (140 respondents were excluded on these grounds). Respondents reported their background and citizen status when entering the panel, so these data were drawn from wave 1, 3, 4, and 5. In addition, data on the items covering stereotypes and emotional prejudices were missing completely (*n* = 32) or largely (>50%, *n* = 22) for some respondents. These were also excluded, leaving a sample size of *N* = 1,062. Thus, respondents who answered 50% or more of the stereotype and prejudice items were retained in the sample, but ignored in the analyses when their response was missing on the variables involved.

In the sample, 51.4% were men. The respondents’ year of birth was pulled from the Population Registry. For anonymity purposes, year of birth is reported in decades in the NCP data. The cohort distribution in the sample was: Born in 1939 or earlier (3.2%), 1940–1949 (19.8%), 1950–1959 (22.7%), 1960–1969 (19.2%), 1970–1979 (17.6%), 1980–1989 (12.2%), and 1990 or later (5.3%). The majority of the sample had some college/university education (58.8%), or had completed high school (27.5%). A minority indicated no education or completed elementary school (9.4%), or declined to indicate their educational level (4.3%). As described above, all participants indicated being Norwegian citizens, born in Norway to Norwegian parents.

### Measures

#### Stereotypes

Following Cuddy et al. (2007), respondents in the collective condition were asked to “*Think about how asylum seekers are viewed by people in Norway in general. In the view of people in general, to what extent are asylum seekers*:” Respondents in the individual condition read: “*Think about how you personally view asylum seekers. In your own view, to what extent are asylum seekers*:” These instructions were immediately followed by one item for competence and one item for warmth “*Competent (capable, confident, and skillful)*” and “*Warm (friendly, good natured, and honest)*”. These items were responded to on a scale from 1 (Not at all) to 5 (To a very large extent).

#### Emotional Prejudices

Participants in the collective condition read: “*Now there will be some questions about what people in general feel about asylum seekers. To what extent do people have the following emotions about asylum seekers as a group?*” Participants in the individual condition read: “*Now there will be some questions about what you personally feel about asylum seekers. To what extent do you have the following emotions about asylum seekers as a group?*” The instructions were followed by a list of emotions: contempt, disgust, admiration, pride, pity, sympathy, envy, jealousy, anger, fear, and anxiety. Although not addressed in the hypotheses, envy and jealousy are included for completeness. These items were also responded to on a scale from 1 (Not at all) to 5 (To a very large extent).

#### Presence of ASC in the Local Community

Respondents read the introduction “*In the last year, a number of new asylum seekers centers have been established in Norway. Do you have an asylum seekers center close to where you live?*” Response categories were “No,” “Yes, my neighborhood has received a new asylum center in the last year,” “Yes, my neighborhood has had an asylum center for more than a year,” and “Do not know.” In this paper, I focus on the subset of respondents (*n* = 228) who had an ASC established in their local community during the last year (i.e., in response to the refugee crisis).

#### Behaviors Directed at the ASC and Asylum Seekers

The instructions to the behavioral items read: “*People have responded to the establishment of ASCs in their local community in different ways. How have you behaved in response to the establishment of an ASC close to where you live? Please indicate how well or how poorly the following statements describe the way you personally have responded. I have…*.” Participants were then presented with a list of items including both facilitating (e.g., participated in voluntary work to help the asylum seekers) and harming (e.g., participated in protests to stop the ASC being set up) behaviors. These items were developed to cover both harming and facilitating behaviors of varying intensities (Cuddy et al., 2007). They were informed by conversations with sociologist Susanne Bygnes, who was conducting qualitative interviews and field work on ASC establishment in local communities in Norway at the time the survey items were created (Bygnes, 2020). Response categories ranged on a five-point scale from “fits very poorly” to “fits very well,” in addition there was a “Not relevant” option (e.g., for people without social media profiles, protesting on social media may be seen as not relevant). In the analyses, “not relevant” was coded as missing.

As the behavioral items had not been employed in previous research, I conducted a principal component analysis. This revealed two components with eigenvalues >1 (eigenvalues were 5.28 and 3.03 for the first and second factors, respectively), which together explained 63.9% of the variance. After rotation (varimax), the two components reflected facilitating and harming intergroup behaviors. The list of items is included in Table 1. Based on this analysis, two intergroup behaviors scales were crated: facilitation (seven items, Cronbach’s *α* = 0.90, *M* = 2.08, *SD* = 1.06) and harm (six items, Cronbach’s α = 0.80, *M* = 1.66, *SD* = 0.75).

## Results

### Stereotype Content and Emotional Prejudice

To address the three hypotheses, a series of one-way ANOVAs were conducted, with a Bonferroni adjusted threshold for significance at 0.0045. Full results are presented in Table 2.

**Table 2:** Means, standard deviations, and one-way analysis of variance for the effects of response instruction on stereotypes and emotional prejudices.

	Experimental group							
	Collective		Individual					
	*M*	*SD*	*M*	*SD*	*F*	*df*	*p*	**η^2^**
Warmth	2.84	0.73	3.25	0.81	75.02	(1, 1,042)	0.000	0.067
Competence	2.66	0.74	3.16	0.80	111.23	(1, 1,048)	0.000	0.096
Contempt	2.70	0.80	1.48	0.79	621.98	(1, 1,056)	0.000	0.371
Disgust	2.54	0.79	1.39	0.71	614.59	(1, 1,056)	0.000	0.368
Anger	2.67	0.88	1.29	0.64	838.88	(1, 1,056)	0.000	0.443
Fear	3.24	0.87	1.85	0.96	611.25	(1, 1,057)	0.000	0.366
Anxiety	3.26	0.84	2.01	0.98	505.98	(1, 1,059)	0.000	0.323
Admiration	2.19	0.72	2.53	0.94	43.83	(1, 1,048)	0.000	0.040
Pride	2.10	0.74	2.10	0.90	0.00	(1, 1,033)	0.999	0.000
Pity	3.31	0.71	3.33	0.93	0.13	(1, 1,057)	0.723	0.000
Sympathy	3.22	0.66	3.43	0.90	19.37	(1, 1,058)	0.000	0.018

Supporting hypothesis 1, stereotypes of asylum seekers were significantly more positive when assessed as individual beliefs (*M_warmth_* = 3.25, *SD* = 0.81; *M_competence_* = 3.16, *SD* = 0.80) than as collective representations (*M_warmth_* = 2.84, *SD* = 0.73; *M_competence_* = 2.66, *SD* = 0.74).

Supporting hypothesis 2, asylum seekers as a group elicited significantly stronger negative emptions in the collective than the individual condition; contempt (*M* = 2.70 vs. *M* = 1.48) disgust (*M* = 2.54 vs. *M* = 1.39), anger (*M* = 2.67 vs. *M* = 1.29), fear (*M* = 3.24 vs. *M* = 1.85) and anxiety (*M* = 3.26 vs. *M* = 2.01). In partial support of hypothesis 3, asylum seekers as a group did elicit more admiration (*M* = 2.53 vs. *M* = 2.19) and sympathy (*M* = 3.43 vs. 3.22) in the individual than the collective condition. The means for pride and pity did not differ significantly.

**Figure 1:**
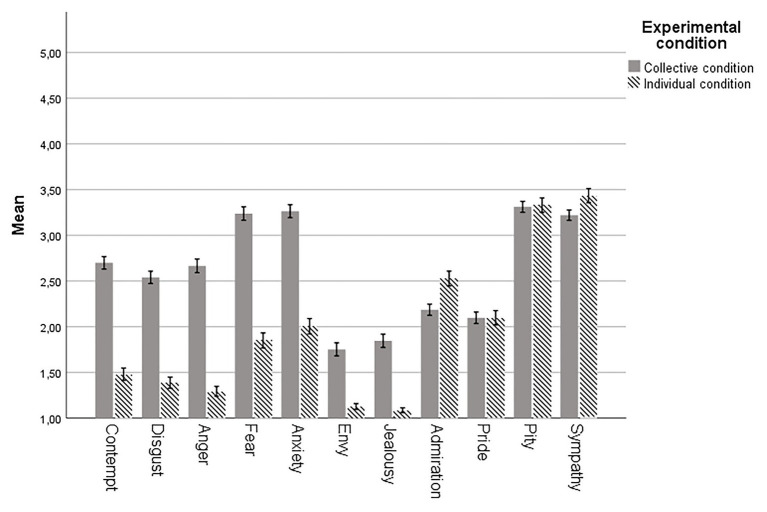
Mean emotional prejudice by experimental condition. Error bars indicate 95% confidence intervals.

As illustrated in Figure 1 (which for completeness includes also envy and jealousy), the differences between individual and group emotional prejudices show a very clear pattern. Across all the negative emotions, the emotional prejudices of others were rated as substantially stronger than the individual emotions. The pattern for the positive emotions, however, is one of similarity. Despite the difference in means between the individual and the collective conditions being statistically significant for admiration and sympathy, these differences were smaller in magnitude than the differences across the negative emotions.

Figure 1 also shows that the mean perception of others’ emotions follows the profile of the mean individually felt emotions very closely. In other words, despite the gap in the reported intensity of the negative emotions, the patterns of the means align.

### Behavior Targeting Asylum Seekers

For those respondents who had received an ASC in their neighborhood in the last year (*n* = 100 and *n* = 128 from the collective and individual conditions, respectively), I correlated ratings of stereotypes and emotions with their reports of facilitating and harming behaviors targeting the newly arrived asylum seekers (Table 3). Recall that the behavioral items all assessed the individual’s own behavior.

**Table 3:** Correlations between stereotypes, emotions and individual behaviors directed at asylum seekers by experimental condition.

	Experimental group			
	Collective condition		Individual condition	
	**Facilitation**	**Harm**	**Facilitation**	**Harm**
Warmth	0.04 [−0.17, 0.25]	−0.24^*^[−0.42, −0.04]	0.32^**^[0.13, 0.49]	−0.38^**^[−0.53, −0.21]
Competence	0.01[−0.20, 0.22]	−0.25^*^[−0.43, −0.05]	0.20^†^[0.00, 0.38]	−0.41^**^[−0.55, −0.24]
Contempt	0.03[−0.17, 0.23]	−0.11[−0.31, 0.09]	−0.21^*^[−0.39, −0.01]	0.27^**^[0.09, 0.43]
Disgust	0.04[−0.17, 0.25]	−0.02[−0.22, 0.18]	−0.20^†^[−0.38, 0.00]	0.27^**^[0.09, 0.43]
Envy	0.03[−0.18, 0.24]	0.04[−0.16, 0.24]	−0.10[−0.29, 0.10]	0.17^†^[−0.01, 0.34]
Jealousy	0.02[−0.19, 0.23]	−0.09[−0.29, 0.11]	−0.01[−0.20, 0.18]	0.16^†^[−0.02, 0.33]
Anger	−0.03[−0.24, 0.18]	0.24^*^[0.04, 0.42]	−0.14[−0.33, 0.06]	0.33^**^[0.16, 0.48]
Fear	−0.12[−0.32, 0.09]	0.18^†^[−0.02, 0.37]	−0.24^*^[−0.42, −0.05]	0.27^**^ [0.09, 0.43]
Anxiety	−0.19^†^[−0.39,0.02]	0.16 [−0.04, 0.35]	−0.19^†^[−0.37, 0.00]	0.25^*^[0.07, 0.41]
Admiration	0.02[−0.19, 0.23]	−0.04[−0.24, 0.17]	0.18^†^[−0.02, 0.36]	−0.44^**^[−0.58, −0.28]
Pride	0.09[−0.13, 0.30]	−0.01[−0.21, 0.20]	0.24^*^[0.04, 0.42]	−0.25^*^[−0.42, −0.07]
Pity	−0.05 [−0.26, 0.16]	−0.04[−0.24, 0.16]	0.09[−0.11, 0.28]	−0.20^*^[−0.37, −0.02]
Sympathy	−0.18 [−0.38, 0.03]	−0.10[−0.30,0.11]	0.17^†^[−0.03, 0.35]	−0.49^***^[−0.62, −0.34]

Participants with a new ASC in the local community (*n* = 228). Sample sizes for the correlational analyses vary from *n* = 85 to *n* = 94 in the collective condition, and *n* = 99 to *n* = 115 in the individual condition. Numbers in square brackets indicate 95% confidence intervals.

*** *p* < 0.001

** *p* < 0.01

* *p* < 0.05

† *p* < 0.10.

Respondents’ perceptions of others’ stereotypes and emotional prejudices were mostly uncorrelated with their own intergroup behaviors directed at the asylum seekers and the ASC in their neighborhood, with some notable exceptions. Perceptions of others’ view on the warmth and competence of asylum seekers correlated negatively with harming behaviors, whereas the perceived anger and fear of others correlated positively with harm. Higher perceived levels of anxiety felt by others correlated negatively with facilitating behaviors.

For respondents who reported their individual stereotypes and emotional prejudices, the correlations revealed a consistent picture in line with the SCM and BIAS Map general predictions. Warmth and competence perceptions correlated positively with facilitation and negatively with harm. Feelings of contempt, disgust, fear, and anxiety correlated negatively with facilitation, and along with anger, envy and jealousy, they correlated positively with harm. Feelings of admiration, pride, and sympathy with asylum seekers correlated positively with facilitation and negatively with harming behaviors. Pity also correlated negatively with harm.

## Discussion

The purpose of this study was to investigate Norwegians’ stereotypes, emotional prejudices, and intergroup behaviors toward asylum seekers following the 2015 refugee crisis and influx of asylum seekers to the country. I also aimed to contribute to the reemerging methodological debate concerning the measurement of stereotypes and prejudices as individual and collective phenomena. Consistent with the first hypothesis, asylum seekers as a group were rated as warmer and more competent when respondents provided their own view as compared to the perceived perspective of others in general. This finding mirrors the results of previous studies of stereotypes of refugees and asylum seekers in Germany (Kotzur et al., 2017, 2019a,b; Froehlich and Schulte, 2019; Wyszynski et al., 2020). It is also consistent with Kotzur et al. (2020) assertion that groups that are deprecated on both the warmth and competence dimension when respondents are instructed to take the perspective of others are rated more positively on both dimensions under instructions to provide one’s personal view. Extending the work of Kotzur et al. (2020), I also found that asylum seekers as a group elicited more contempt, disgust, anger, fear, and anxiety in the collective condition than when respondents reported their individual emotions. When reporting their own emotions, respondents indicated more admiration and sympathy for asylum seekers than they perceived others to experience. These differences were small, however.

The differences across conditions in the reported emotional prejudices were in line with the hypotheses and consistent with the tenets of the SCM and the BIAS Map that emotional prejudices follow from stereotype content (Fiske et al., 2002; Cuddy et al., 2007). However, it is noteworthy that the differences across conditions in the negative emotions were considerably stronger than the differences in stereotype content and positive emotions (see effect sizes in Table 2). Although the sample in this study was not perfectly representative of the Norwegian population, it does provide a close approximation. We can therefore regard the average responses in the individual condition as an approximation of the “correct answer” to the question posed in the collective condition of what people in Norway in general think and feel about asylum seekers. When considered from this perspective, the results of the experiment tell us at least two things. First, the average perception of others’ stereotypes and positive emotions appear to be fairly accurate, at least in the case of asylum seekers as target group, despite individual ratings being somewhat more positive. Second, the perception of others’ negative emotions is accurate in terms of the pattern of emotions, but not in the average intensity. I cannot rule out that individuals underreported the intensity of their personal negative emotions due to social desirability concerns. However, there is also a possible substantive interpretation. Negative emotions associated with asylum seekers may have been perceived to be stronger among other people in general as a consequence of the intense media focus and a national political debate centered on restricting arrivals to the country (Bygnes, 2020).

In line with this interpretation of the findings, Gaucher et al. (2018) found in a longitudinal study that warmth and competence stereotypes of migrants and refugees (rated from the perspective of “most Canadians”) became more positive following a change in government and related changes in political rhetoric and policies toward refugees. This change was stronger among individuals motivated to justify their sociopolitical system. In other words, the perceived stereotypes of others toward refugees were influenced by changes in the government policy, and the same type of process may be operating with respect to the perception of negative emotions targeting asylum seekers. This interpretation is also in line with the claim that “people’s understanding of culturally shared stereotypes takes the perspective of society’s dominant reference groups.” (Fiske et al., 2002, p. 881), which in times of intense political debate and news coverage may be politicians at the national stage.

Among the subgroup of the respondents who had had an ASC established in their neighborhood in the last year, the results showed that individual stereotypes and emotional prejudices were consistently correlated with individual harm and facilitation toward asylum seekers in line with SCM and BIAS Map predictions. The pattern of correlations was less consistent when individual intergroup behaviors were correlated with perceptions of collective stereotypes and prejudices. However, the perception that others hold more negative stereotypes of asylum seekers, and the perceived anger and fear of others, did correlate with individual harming behaviors. Perceptions of others’ anxiety correlated negatively with facilitating behaviors. Whereas the interpretation of the stereotypes–emotions–behavior correlations is uncomplicated in the case of the individual response instructions, and the relationships between the perceived stereotypes and emotions of others and individual behaviors require more discussion.

The pattern of correlations in the collective condition could be interpreted as reflecting the impact of perceived descriptive norms of stereotypes and emotions toward asylum seekers in society on individuals’ self-reported behaviors, in line with Cuddy et al.’s (2007) argument that knowledge of cultural stereotypes and prejudices impact individuals’ behaviors. It could also be interpreted within an intergroup emotions framework (Mackie and Smith, 2015). Intergroup emotion theory explicitly emphasizes the role of self-categorization processes in emotion, reserving the group emotion term for “emotions that causally depend on self-categorization, that occur whether or not the group is physically present, and that reflects group-level, rather than interpersonal processes” (Mackie and Smith, 2015, p. 264). Although the respondents were not explicitly asked to think of themselves as Norwegians in the collective condition, the instruction to indicate the view of people in Norway in general, may have increased the salience of this group membership. There results from the collective condition are consistent with both these perspectives, so disentangling them would require additional studies.

Kotzur et al. (2020) argued that due to the differences they observed between the individual and collective response instructions on stereotype ratings, the way forward is to ask individuals to provide their personal views and aggregate these to the cultural level. Although this may very well be a valid approach to mapping the stereotype content of social groups within and across countries, it is important to acknowledge that stereotype ratings gathered from a collective approach have been related to cultural values, economic indicators and other country-level factors (Durante et al., 2013, 2017; Cuddy et al., 2015). Another approach put forward by Findor et al. (2020) is to treat individual and collective stereotypes and prejudices as separate constructs. They argue that individual instructions are adequate for assessing individuals’ stereotypes and prejudices and they suggest interpreting collective stereotypes as indicators of the normative context or climate in a society. Thus, it appears premature to abandon the collective approach to the measurement of stereotypes and emotional prejudices. Rather, I would argue that systematically combining and comparing them could be a way toward new insights, as a number of questions remain unanswered. What are the relationships between the perceived stereotypes and emotional prejudices of others in society and individually held stereotypes and emotions? For example, if the perceived stereotypes of others in society toward politicized groups (e.g., refugees) changes with political rhetoric and policy, as indicated by the work of Gaucher et al. (2018), is this change also reflected in individuals’ personal perceptions? In this study, I found that both individuals’ personally held stereotypes and prejudices and perceptions of others’ stereotypes and emotions (anger, fear, and anxiety) were associated with intergroup behaviors. However, because of the between-groups design, I could not compare their relative contributions to intergroup behaviors within individuals. Addressing this issue with a different design would be a valuable direction for future research.

### Strengths and Limitations

The present study has a number of strengths. An experimental design and a large sample from the Norwegian adult population provides a solid foundation for comparing the impact of response instructions on ratings of stereotypes and emotional prejudices. However, there are several limitations that need to be addressed. The measures of stereotypes were based on single items, which prevented the latent variable modeling advocated by other stereotype researchers (e.g., Kotzur et al., 2020). With respect to the relationships between the stereotype and emotional prejudice ratings and the intergroup behaviors, the data were collected at the same time, and the cross-sectional and correlational design limits causal inferences.

Because I wanted to study intergroup behaviors among respondents who had recently had asylum seekers move in to ASCs in their neighborhood, the sample size for the intergroup behaviors analyses were smaller than might be desired. The *n* for the subsample correlations between stereotypes and prejudices and intergroup behaviors varied, with the smallest *n* = 85. With that sample size (given *α* = 0.05, two-tailed test), I had a power of 0.99 to detect a population correlation of 0.50, a power of 0.81 to detect a population correlation of 0.30, and a power of 0.15 to detect a population correlation of 0.10 (G*Power version 3.1.9.4; Faul et al., 2007; O’Keefe, 2007). Small-to-moderate correlations between stereotypes and emotional prejudices on the one hand and intergroup behaviors on the other may be both theoretically and practically relevant. It is important to acknowledge that the size the subsample in the present study does not allow for establishing the existence of smaller effects.

Although the behaviors included in the intergroup behavior items were intended to capture both active (e.g., befriending) and more passive (e.g., commenting on social media) behaviors, the principal component analysis indicated only two components capturing facilitating and harming behaviors in general, without separating them along the active-passive dimension (Cuddy et al., 2007). This prevents a formal test of the mediation hypotheses central to the BIAS map. It is also important to note that the means of both harming and facilitating behaviors were low and the distributions of responses were skewed, especially for harming behaviors. From a substantive perspective, this indicates that several respondents reported not having engaged in the behaviors depicted in the items. This it interesting in itself, as it suggests that having an ASC established in the neighborhood triggers less backlash, but also less prosocial responses, than might have been expected (see also Bygnes, 2020). From a methodological perspective, the skewed distributions could have attenuated the correlations between the intergroup behaviors and the stereotype and prejudice measures.

## Conclusion

I found that following the 2015 refugee influx to the country, Norwegians’ stereotypes of asylum seekers centered on ascriptions of moderate warmth and competence – perceptions were more positive when respondents provided their personal views, and more negative when reporting their perceptions of the views of other people. Across response instructions, the emotional responses to asylum seekers as a group were characterized by pity and sympathy. When reporting the perceived emotions of others, asylum seekers were also perceived to elicit fear and anxiety, and to a certain extent anger, contempt and disgust. Among individuals who had a reception center for asylum seekers established in their neighborhood during the crisis, individual stereotypes and prejudices, and the perceived stereotypes and prejudices of others, were related to facilitating and harming intergroup behaviors. The average reported levels of both harming and facilitating behaviors were low, however. This may suggest that for a majority of individuals in receiving communities, hosting asylum seekers in an ASC in the neighborhood elicited neither backlash nor a strong prosocial behavioral response.

I found that response instructions impacted both the reported stereotype content and emotional prejudices, but do not conclude that one approach should be preferred over the other. Rather, I argue that systematically combining and comparing them could be a way toward new insights, as a number of questions remain unanswered.

## Data Availability Statement

The datasets presented in this study can be found in online repositories. The names of the repository/repositories and accession number(s) can be found below: the data applied in the analysis in this publication are based on the Norwegian Citizen Panel [Waves 1 (2013), 3 (2014), 4 (2015), 5 (2015), and 6 (2016)]. The data are provided by UiB, prepared and made available by Ideas2Evidence, and distributed by the Norwegian Centre for Research Data (NSD). Neither UiB nor NSD are responsible for the analyses/interpretation of the data presented here. Data from the Norwegian Citizen Panel are available upon request from the Norwegian Centre for Research Data: https://nsd.no/nsddata/serier/norsk_medborgerpanel.html. Data from the Norwegian Citizen Panel are available for non commercial use. Please see conditions of use here: https://www.uib.no/en/digsscore/122158/data-and-conditions-use.

## Ethics Statement

The studies involving human participants were reviewed and approved by The Norwegian Data Protection Authority (License number 34817). The patients/participants provided their written informed consent to participate in this study.

## Author Contributions

HHB designed the study, analyzed the data, and wrote the manuscript.

### Conflict of Interest

The author declares that the research was conducted in the absence of any commercial or financial relationships that could be construed as a potential conflict of interest.

## Footnotes

^1^“As viewed by society, does this group make your group feel: disappointed, fearful, sympathetic…”(Fiske et al., 2002, p. 896). “Now I’m going to ask you about some feelings that people in America have towards (group) as a group. To what extent do people tend to feel (emotion, e.g., pity) towards (group)?” (Cuddy et al., 2007, p. 648).
